# The Burden of the Serious and Difficult-to-Treat Infections and a New Antibiotic Available: Cefiderocol

**DOI:** 10.3389/fphar.2020.578823

**Published:** 2021-01-14

**Authors:** Yasaman Taheri, Nataša Joković, Jelena Vitorović, Oliver Grundmann, Alfred Maroyi, Daniela Calina

**Affiliations:** ^1^ Phytochemistry Research Center, Shahid Beheshti University of Medical Sciences, Tehran, Iran; ^2^ Department of Pharmacology and Toxicology, School of Pharmacy, Shahid Beheshti University of Medical Sciences, Tehran, Iran; ^3^ The Faculty of Science and Mathematics, University of Niš, Niš, Serbia; ^4^ Department of Medicinal Chemistry, College of Pharmacy, University of Florida, Gainesville, FL, United States; ^5^ Department of Biobehavioral Nursing Science, College of Nursing, University of Florida, Gainesville, FL, United States; ^6^ Department of Botany, University of Fort Hare, Alice, South Africa; ^7^ Department of Clinical Pharmacy, University of Medicine and Pharmacy of Craiova, Craiova, Romania

**Keywords:** antimicrobial treatment, new antibiotic, cefiderocol, siderophore cephalosporin, multi drug-resistant gram-negative bacilli, critically ill patients, bacterial severe infections

## Abstract

**Background:** Infection is a disease that can occur due to the entrance of a virus, bacteria, and other infectious agents. Cefiderocol is innovative cephalosporin drug that belongs to a special class of antibiotics, sideromycins, which are taken up by bacterial cells through active transport. The unique cell entry and stability to β-lactamases allow cefiderocol to overcome the most common resistance mechanisms in Gram-negative bacteria.

**Objective:** This article aims to highlight the therapeutic efficacy, safety and tolerability of cefiderocol, with a focus on the FDA label.

**Methods:** The pharmacological properties of cefiderocol are also summarized. In this review, we conducted literature research on the PubMed database using the following keywords: “antimicrobial treatment”, “new antibiotic”, “cefiderocol”, “siderophore cephalosporin”; “multidrug-resistant”, “Gram-negative bacilli”, “critically ill patients”; “severe bacterial infections”.

**Results:** There were identified the most relevant data about the pathophysiology of serious bacterial infections, antibacterial mechanism of action, microbiology, mechanisms of resistance, pharmacokinetic and pharmacodynamic properties of cefiderocol.

**Conclusion:** The results highlighted there appeared to be clinical benefit from cefiderocol in the treatment of infections caused by Gram-negative aerobic microorganisms in adult patients with severe infections and limited treatment options.

## Introduction

Infectious diseases have been and remain a significant cause of death, disability, and social and economic costs for individuals and the healthcare system. Until the end of the twentieth century, infectious diseases were the main responsible for the most significant global burden of disability and premature death. However, infectious diseases remain a threat to human life because of the possibility of outbreaks and also the rise of antimicrobial resistance due to increased use of antibiotics (Salehi et al., 2014; Sharifi-Rad et al., 2018a; Sharifi-Rad et al., 2018b; Mishra et al., 2018; Salehi et al., 2018). The mortality rate of infectious diseases has had a declining trend worldwide because of an improvement in quality and access to healthcare services and prevention measures such as vaccines (El Bcheraoui et al., 2018).

The rise of antimicrobial resistance is a challenge for the treatment of infectious diseases. There is an increasing concern with antimicrobial resistance (AMR) across countries as a result of inappropriate use of antibiotics increasing morbidity, mortality and costs (O’Neill, 2014; Jinks et al., 2016; Founou et al., 2017; Cassini et al., 2019; Hofer, 2019).

Urinary tract infections (UTIs) are bacterial infections of the bladder and the associated parts. Uncomplicated UTI, also known as Cystitis or lower UTI, is an infection in patients without any other complications such as diabetes, pregnancy, immunocompromised, etc. The uncomplicated cases are mostly self-limited. The goal of treatment in these infections is to limit the infection spread to the kidneys or to grow into pyelonephritis. There is the ongoing controversy surrounding the routine use of antibiotics for routine use in uncomplicated UTIs—more beneficial in the elderly (Kronenberg et al., 2017; Gharbi et al., 2019). which is why UTIs are often used in simulated client projects to assess the rate of self-purchasing of antibiotics (Alrasheedy et al., 2020).

**FIGURE 1:**
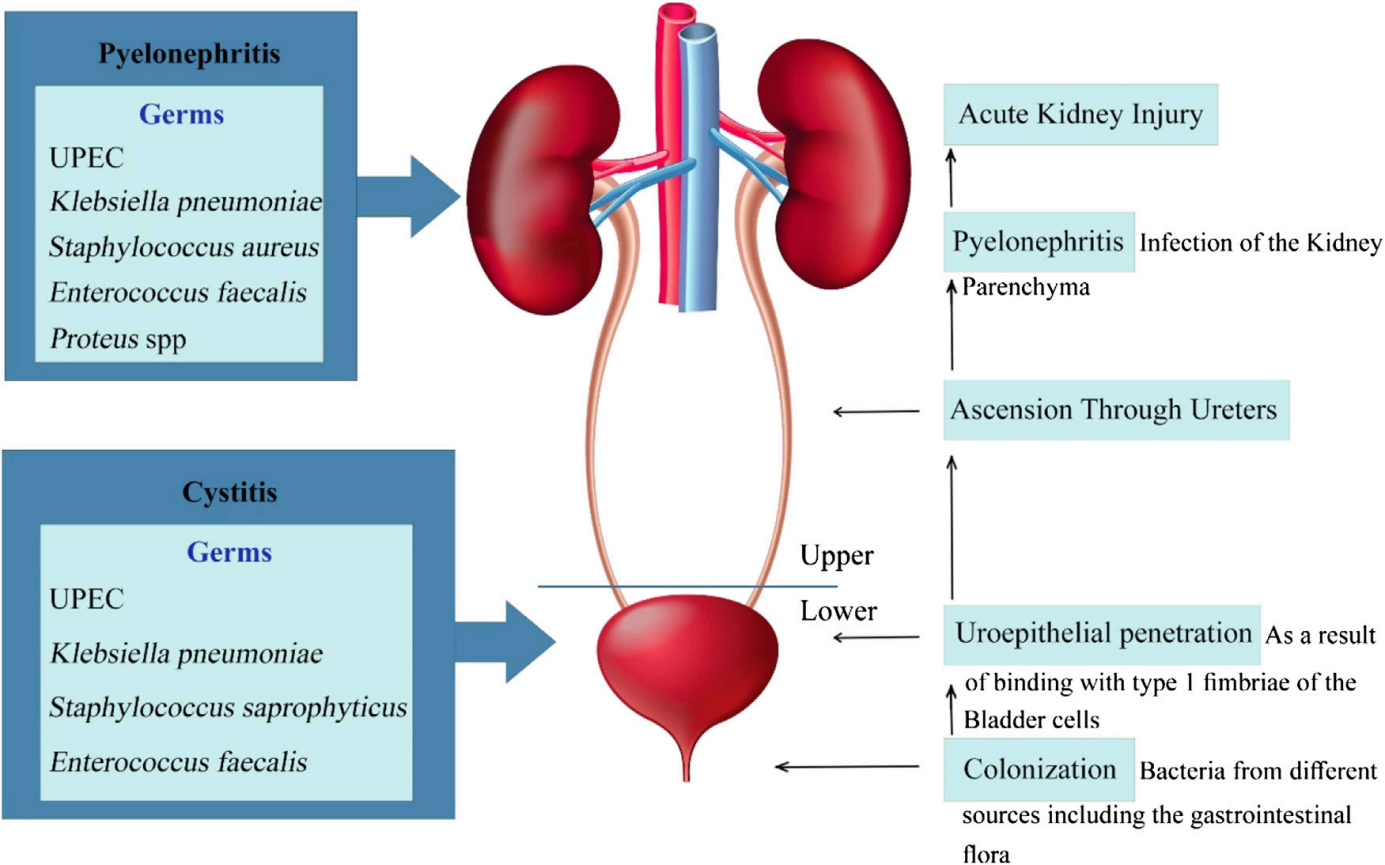
Schematic presentation of the pathophysiology of uncomplicated and complicated urinary tract infections.

Complicated urinary tract infection and pyelonephritis are also common genitourinary infections. Complicated UTI is localized in the lower and upper urinary tract and is due to structural or functional abnormalities and may have the risk of treatment strategy failure and develop pyelonephritis, obstruction in the urinary tract and bladder and kidneys dysfunction (Manosuthi and Wiboonchutikul, 2016). (Figure 1).

One of the most significant threats in global health is the carbapenem-resistant gram-negative bacteria and lack of efficient antibiotics. The world health organization designated carbapenem-resistant Enterobacteriaceae, *Pseudomonas aeruginosa*, and *Acinetobacter baumannii* as high-priority pathogens, which urgently need new antimicrobial development (Yamano, 2019; Alrasheedy et al., 2020).

In 2010, the Infectious Diseases Society of America (ISDA) launched policy recommendations to combat the crisis of antimicrobial resistance. The strategies recommended, call for the development of 10 novel, safe, and effective systemic antibiotics by 2020, particularly those with an effect against gram-negative infections (America, 2011).

On November 14, 2019, cefidrocol, a novel siderophore cephalosporin, received the U.S. Food and Drug Administrations (FDA) approval, for the treatment of adults (patients 18 years of age or older) with complicated urinary tract infections (cUTIs) for, including kidney infections caused by susceptible Gram-negative microorganisms, who have limited or no treatment options (McCarthy, 2020).

This study aims to provide information on antimicrobial-resistant infections with a focus on complicated urinary tract infections, and the analysis of cefiderocol as an option for patients with multidrug-resistant Gram-negative bacterial infections.

## Methodology

The literature available in the Pubmed database was researched for therapeutic efficacy, dosage and administration and tolerability of cefiderocol in serious and difficult-to-treat infections by applying the following keywords: “antimicrobial treatment”, “new antibiotic”, “cefiderocol”, “siderophore cephalosporin”; “multidrug-resistant”, “Gram-negative bacilli”, “critically ill patients”; “severe bacterial infections” and their titles corresponding to the medical subject (MeSH) using OR/AND conjunctions. The research focused on cefiderocol and its clinical implications. The searches were limited to published papers in English and did not include studies with homoeopathic preparations.

## Results

### Pathophysiology of Serious Bacterial Infections: A Brief Overview

**FIGURE 2:**
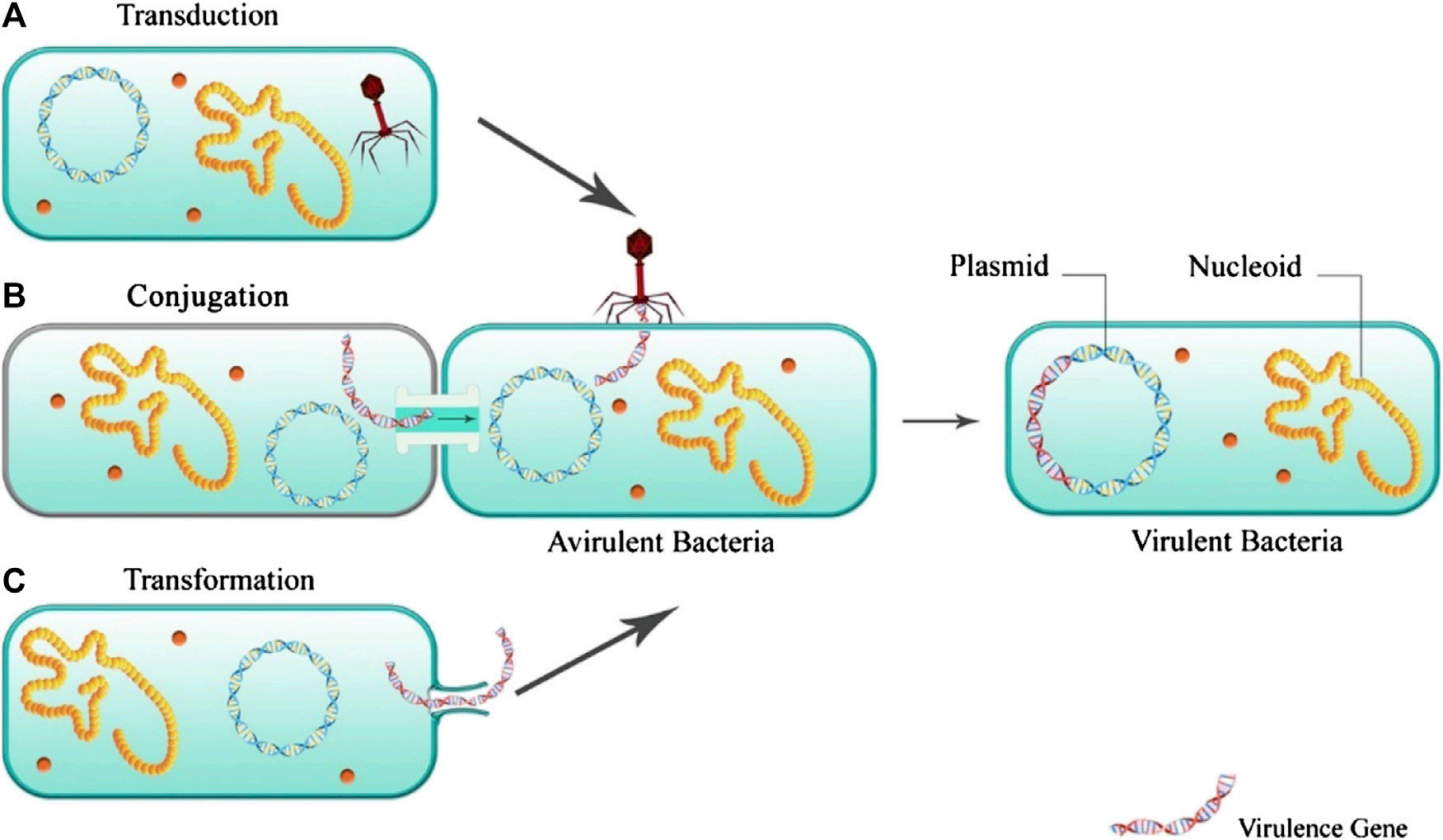
Acquiring virulence genes by the bacteria.

Infection occurs when the balance between the pathogenicity of the virulence factor and the host immunity is upset. Virulence factors of bacterial infections include toxins, surface coats that inhibit phagocytosis, and receptors that bind to the host cell. Many virulence factors are explicitly produced by strains of microorganisms. Factors such as high mutation rate and rapid generation of the bacteria, resulting in the selection of the best-adapted microorganisms (Murray et al., 2015). The virulence factors of bacteria are based and encoded in their DNA, bacteriophage DNA, plasmids, and transposons. Figure 2 illustrates the mechanisms of acquiring virulence genes by the bacteria.

Among transferring the virulence genes, the acquisition of the antibiotic resistance gene is one of the most significant medical challenges. This can provide the chance for the resistant bacteria for the generation and development of more virulence factors, especially in patients receiving an inappropriate antibiotic-based treatment (Escudeiro et al., 2019).

The bacterial toxin promotes infection by damaging the host cells (Ungureanu et al., 2017). The exact function of the toxin to the bacterium is unknown. Some of the protein toxins are enzymes. Nevertheless, in many cases, the purpose of the toxins is unknown. However, it is highly unlikely for the bacterium to spend the energy for producing a complex and high-molecular-weight molecule if they do not serve it any advantages (Murray et al., 2015).

Based on the invasion mechanism to the eukaryotic cells, the bacteria are divided into two groups of extracellular bacteria that survive as free-living pathogens in their environment, and the intracellular bacteria which exist and replicate inside the host cells. Facultative intracellular bacteria can survive inside in either intra- and extracellular environments. On the contrary, obligate intracellular bacteria only require the host cell environment for survival and replication (McClure et al., 2017).

**FIGURE 3:**
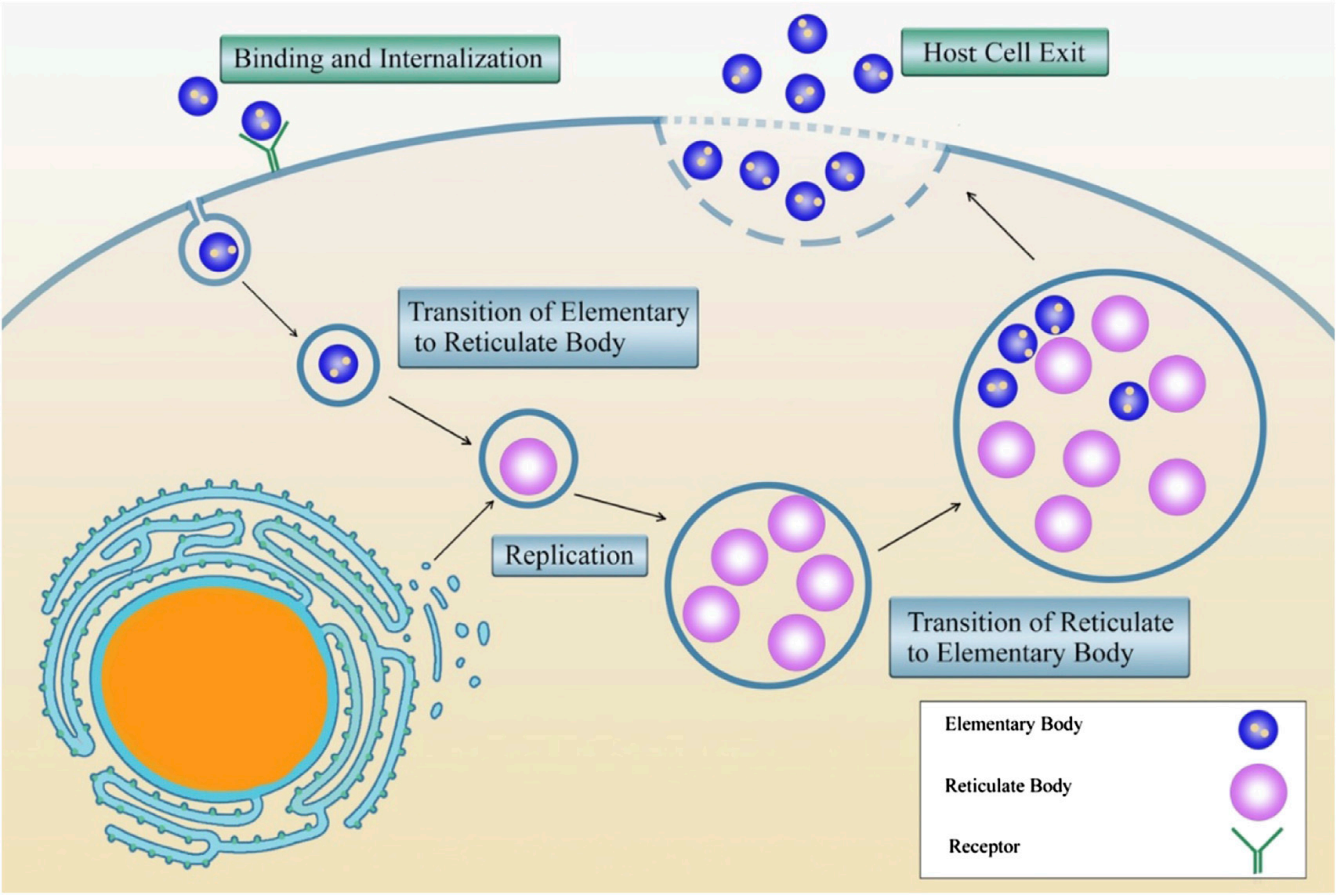
The life cycle of *Chlamydia* spp. as an example of obligate intracellular bacteria.

Intracellular bacteria promote the ability to enter the host cell and resist the cellular antimicrobial mechanisms (Călina et al., 2017). X After this stage, the bacteria modulate the eukaryote cell biology to develop a novel intracellular replication setting (McClure et al., 2017). Figure 3 shows the life cycle of *Chlamydia* spp. as an example of the obligate intracellular life cycle and replication.

**FIGURE 4:**
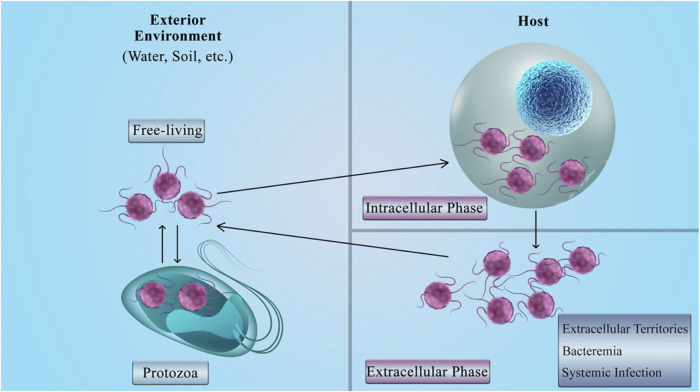
Facultative bacteria life cycle.

The facultative intracellular bacteria have the capability to lead a dual intracellular/extracellular lifestyle (Tanase et al., 2016). This means that not only these bacteria have the intracellular growth phase, but also they can survive in the extracellular and natural settings like in the host's environment as free-living bacteria (Silva and Pestana, 2013; Călina et al., 2016). The bacteria can enter the eukaryotic cells and survive by using their resources. From another point of view, entering the host cells can shield the bacteria from the antibodies; as a result, only the cellular immune system can eliminate this invasion (McClure et al., 2017). Figure 4 presents a brief explanation for the life cycle of facultative intracellular bacteria.

Most bacteria proliferate in the extracellular environment. Moreover, some do not even transude the body tissue; instead, they adhere to the epithelial and cause infection by producing potent toxins. Bacteria such as *E. coli* and *P. aeruginosa* in UTI once gain access to the body, can frequently generate and spread through the body tissues (Murray et al., 2015).

A single microorganism mainly causes uncomplicated UTI. Uropathogenic *E. coli* (UPEC) is responsible for more than 80% of the Urinary Tract community-acquired infections. Among staphylococci, *S. saprophyticus* is the most common cause of uncomplicated UTI. *S. saprophyticus* is present in 5% to more than 20% of cases of uncomplicated UTI (Floege et al., 2010). Other bacteria include *Enterobacter*, *Klebsiella*, *Staphylococcus*, *Proteus*, and *Enterococcus,* which mostly cause complicated, catheter-associated, and hospital-acquired infections. These infections can be polymicrobial (in 30% of cases), particularly in the presence of stones. *Pseudomonas aeruginosa* and yeast infection are also present in these infections. There are cases of antimicrobial resistance in many of the bacteria responsible for severe and complicated UTI (Sharifi-Rad et al., 2016).

The uropathogens like UPEC, from different sources including the gastrointestinal flora, access the urethra, ascends along the mucosal sheath, enters the bladder, and binds to the cells (facet cells) in the epithelial surface of the bladder with type 1 (mannose-sensitive) fimbriae (Hannan et al., 2012). After that, the fimbriae are not presented anymore; as a result, it will not bind the Tamm-Horsfall protein and also the immunoglobulin A (IgA) and will prevent recognition by the immune systems phagocytic cells. However, it also possesses type 1 receptor (Floege et al., 2010).

When the bacteria enter the facet cells, the neutralization by the UPEC is sensed by a protein in the lysosomal membrane named mucoplipin TRP channel 3 (TRPML3). The activation of this protein starts a cascade that results in the bladder cells exocytosing the UPEC-containing lysosomes; therefore, the expulsion of intracellular UPEC (Miao et al., 2015). UPEC can form a biofilm on the mucosal surface. They can invade mature and immature cells. Intracellular UPEC can multiply, return to the lumen, or enter the cytoplasm and develop intracellular bacterial community (IBC). They can also produce dormant intracellular reservoirs that have the potential to re-invade. In the meantime, the host facet cells can expel the IBCs to urine. The liberation of IBCs to the urine is detected in the urine samples from the infected patient. This is a hallmark of laboratory findings showing a present infection (Floege et al., 2010).

### Antibacterial Mechanism of Action, Microbiology, Mechanisms of Resistance, Pharmacokinetic and Pharmacodynamic Properties of Cefiderocol

#### Antibacterial Mechanism of Action

The primary mechanism of cefiderocol antibacterial action is a disruption of the bacterial cell walls, as with other cephalosporins, but it has a unique mechanism of penetration in bacterial cells using a Trojan horse strategy by imitating a process that occurs naturally when bacteria are found in the iron-depleted environment. The chlorocatechol group on the end of the C3 side chain of cefiderocol acts as the siderophore that forms a complex with insoluble ferric iron enabling molecule to pass through the outer membrane of Gram-negative bacteria via specific ferric iron transport systems (Ito et al., 2016a). Experiments with mutants have shown that iron transporters PiuA in *Pseudomonas aeruginosa* and CirA and Fiu in *Escherichia coli* are responsible for the active transport of cefiderocol in the bacterial cells, while deficiency of porin OmpK35/36 in *Klebsiella pneumoniae* and the overproduction of multidrug efflux pump MexA-MexB-OprM in *P. aeruginosa* are not significant for this process (Ito et al., 2018c). After entering the periplasmatic space, iron dissociates from siderophore and cephalosporin core of cefiderocol covalently binds to penicillin-binding proteins (PBPs), mainly for PBP3, leading to inhibition of peptidoglycan synthesis in the bacterial cell wall. The antibacterial activity of cefiderocol is further enhanced by a positive charged cyclic quaternary ammonium moiety on the C 3 side chain, similar to cefepime, making it a zwitterion, allowing better orientation of the molecules towards the negatively charged inner membrane of the bacterial cell. The C 7 side chain is the same as ceftazidime with the aminothiazole ring for incrasing binding affinity to PBP and enhancing antibacterial activity, while carboxypropyl-oxy- imino group provides better outer membrane permeability (Sato and Yamawaki, 2019).

#### Microbiology

Cefiderocol has a characteristic antibacterial spectrum with the highest efficiency against Gram-negative bacilli belonging to Enterobacteriaceae and non-fermenting bacilli such as *P. aeruginosa*, *Acinetobacter baumannii* and *Stenotrophomonas maltophilia* including carbapenem-resistant (CR) and multidrug-resistant (MDR) strains (Zhanel et al., 2019). Cefiderocol showed minimal inhibitory concentration (MIC) values ≤ 2 μg/ml against a broad range of Gram-negative bacteria including Enterobacteriaceae (*Enterobacter* spp., *E. coli*, *Klebsiella* spp., *Proteus* spp., *Providencia* spp., *Salmonella* spp., *Yersinia* spp.), *Acinetobacter* spp., *Pseudomonas* spp., *Burkholderia* spp, *Vibrio* spp., *Haemophilus* spp. and *Neisseria* spp. The MICs for a strain of *Neisseria gonorrhoeae* 868339 and two *Campylobacter jejuni* strains were higher than 4 μg/ml. On the other hand, cefiderocol had poor activity against Gram-positive bacteria, except for two strains of *Streptococcus* spp., and anaerobic bacteria due to the lack of the active ferric iron uptake in these bacteria (Ito et al., 2018c).

Cefiderocol is being developed primarily for treating infections caused by MDR bacterial strains, especially CR Gram-negative bacilli that are identified as pathogens of particular concern. Carbapenem resistance occurs in bacteria due to reduction or modification of porins in the outer bacterial membranes, overexpression of efflux pumps, synthesis of antibiotic degrading β lactamases among which ESLBs and carbapenemases are the most important or less frequently, modification of PBPs (Ruppé et al., 2015) The advantage of cefiderocol over other antibiotics is the unique mechanism of entering the cell, which overcomes the resistance mechanisms related to porins and efflux pumps. Additionally, cefiderocol is stable against hydrolytic action of a wide variety of β lactamases including carbapenemases due to a pyrridoline ring bound to the catechol moiety on the C3 chain and oxime and dimethyl group on the C 7 side chain which prevents binding of enzymes for the main core of antibiotic ^3^. Experiments with purified enzyme extracts of KPC-3, IMP-1, VIM-2, NDM-1, L1, OXA-48, OXA-40, OXA-23 β-lactamases revealed that cefiderocol remained stable when exposed to these enzymes (Ito-Horiyama et al., 2016; Poirel et al., 2018). The antibacterial activity of cefiderocol against strains producing extended spectrum β-lactamases (ESBLs) and carbapenemases, such as KPC, NDM, VIM, IMP, OXA-23, OXA-51-like and OXA-58 have been well documented (Wu et al., 2020). Cefiderocol also demonstrated antibacterial activity against AmpC-overproducing strains of *P. aeruginosa* and *Enterobacter cloacae*, and low affinity for chromosomal AmpC β-lactamases, and its low induction (Ito et al., 2018b).

#### Mechanisms of Resistance

Data on the development of cefiderocol resistance in Gram-negative bacteria are very scarce. Frequency of spontaneous mutation in Gram-negative bacilli exposed to a 10-fold higher concentration of cefiderocol than MICs of tested isolates ranged from <7.1 × 10^–9^ to 1.6 × 10^–6^ and was lower than those of ceftazidime (Ito et al., 2016b; Sato and Yamawaki, 2019). Daily serial passage for 10 days increased MICs values of cefiderocol up to 4-fold in 1 MDR *P. aeruginosa* (IMP-1 producer) and 2 KPC-producing *K. pneumoniae* isolates (Kohira et al., 2018). Reduced susceptibility to cefiderocol in strains with evaluated MICs may be due to the presence of β-lactamases, although a clear correlation between β -lactamase production and increased MICs to cefiderocol has not yet been found. Among 72 isolates of *A. baumannii* and Enterobacteriaceae that showed evaluated cefiderocol MICs of ≥8 mg/ml, PER and NDM encoding genes were detected in 32 *A. baumannii* and 15 Enterobacteriaceae, respectively*.*

Addition of avibactam (a serine β-lactamase inhibitor), decreased cefiderocol MICs of PER-producing strains for more than 4-fold. NDA producing strains showed ≥4-fold lower MICs only when combination of avibactam and dipicolinic acid (metallo-β-lactamase inhibitor) were added indicating that resistance mechanism of these strains was due to additional β-lactamases (Laurent et al., 2019). Resistance to cefiderocol may arise from mutations in genes involved in iron uptake. In *P. aeruginosae* PAO1 mutants obtained after exposure to cefiderocol, mutations in promotor regions of *pvdS* (regulates pyoverdine synthesis) and *fecI* (synthesis of iron transporter FecA important for the transport of iron citrate) were detected by whole genome sequencing (WGS). Overexpression of these genes increased MICs to cefiderocol 4-fold (Ito et al., 2018a).

#### Pharmacokinetics

Pharmacokinetic profile of cefiderocol was determined in a single- and multiple-dose studies conducted in healthy subjects and subjects with different degree of renal impairment in phase I clinical studies.

Cefiderocol pharmacokinetics (PK) was linear after intravenous infusion over 60 min of single and multiple doses ranging from 100 to 2 000 mg in healthy subjects and only slightly accumulation in plasma was observed following multiple dosing. A mean plasma half-life (t_1/2_) ranged from 1.98 to 2.74 h, while a total drug clearance (CL) was within range of 4.6 to 6.0 L/h (Saisho et al., 2018).

Detailed metabolic profiling of radioactive labeled [14C]- cefiderocol and its metabolites demonstrated that unchanged cefiderocol was predominant fraction in plasma (92,3%) with a negligible portion in red blood cells. The main excretion pathway of cefiderocol was via kidneys, 98,7% of [14C]- cefiderocol was detected in urine with 90.6% in unchanged form, remaining cefiderocol was found in faeces. Cefiderocol and compounds related to it were fast removed from body for 120 h after initiation of administration with no remaining compounds in body (Miyazaki et al., 2019). To determine how renal function affects the PK of cefiderocol, subjects with mild (eGFR 60 to <90 ml/min/1.73 m^2^), moderate (eGFR 30 to <60 ml/min/1.73 m^2^) and severe (eGFR <30 ml/min/1.73 m^2^) impairment and with end-stage renal disease (ESRD) (eGFR, <15 ml/min/1.73 m^2^) undergoing or not hemodialysis, as well as subjects with normal renal function (eGFR, ≥90 ml/min/1.73 m^2^) were enrolled in a phase I preclinical study. The values of PK parameters, the maximum plasma concentration (C_max_) and plasma-protein-unbound fraction were similar between groups, while the area under the plasma concentration–time curve (AUC), t_1/2_ and CL were dependent on renal function. Additionally, 60% of cefiderocol was excluded by hemodialysis for 3 to 4 h.

The results induced that dose adjustment are needed for patients with renal impairment, while patients receiving intermittent HD need supplemental dose of cefiderocol to achieve cefiderocol concentration similar as in people with normal renal function (Katsube et al., 2017a).

The dose adjustment regimens depending on renal function were predicted by a population pharmacokinetic/pharmacodynamic modeling and simulation using data previously reported. Monte Carlo simulation revealed that cefiderocol regimen 2 g q8h received with 1- or 3-h infusion is effective in achieving bactericidal effect of cefiderocol against bacteria with MIC ≤4 mg/ml. Considering that critically ill patients are target population for cefiderocol treatment and that β-lactam antibiotics are applied through prolonged infusion, infusion of 3 h was chosen for all cefiderocol regiments. A standard dose regimen is 2g q8h with a 3-h infusion for the patients with normal renal function and with mild impairment. Fewer doses of 1.5 and 1 gr were proposed for groups with moderate and severe impairment, respectively. Cefiderocol regimen for ESRD groups and those that requiring intermittent HD is 0.75 g q12h, 3-h infusion. The regiment 2 g q6h, 3-h infusion was proposed for the group with augmented (CG-CLCR, ≥120 ml/min) renal function (Katsube et al., 2017b). PK of cefiderocol wasn’t significantly changed after intravenously infusion of cefiderocol supratherapeutic dose (3and 4 g) for 3 h in healthy subjects. Examination of ECG parameters in healthy subjects who received therapeutic (2 g) and supratherapeutic (3 and 4 g) doses of cefiderocol revealed that QT/QTc interval or other ECG parameters were not affected by the administration of these doses and no clinically significant adverse events (AEs) were observed (Sanabria et al., 2019).

The study of intrapulmonary penetration of cefiderocol showed that cefiderocol PK in epithelial lining fluid (ELF) and plasma were parallel indicating that cefiderocol penetrates fast in ELF which could be further used for prediction of cefiderocol concentration in ELF based on its plasma concentration (Katsube et al., 2019). Cefiderocol also had no clinically significant inhibitory effects on PK of co-administered drugs that are substrates of gut, hepatic, and renal (Katsube et al., 2018a).

#### Pharmacodynamics

The pharmacodynamic parameter that is in the best correlation with the efficiency of cefiderocol *in vivo* is the percent of the time when the free concentration of drug exceeds the MIC (% fT > MIC). This correlation was observed before for the other β-lactam antibiotics and several studies confirmed that % fT > MIC can predict the effectiveness of cefidercol *in vivo*, estimated through the change in the number of bacteria.

Cefepime and cefiderocol tested against *P. aeruginosa* showed the highest *R* ^2^ value (square of the correlation coefficient) of %*fT*MIC among two other tested PK/PD parameters, the free drug concentration in plasma divided by MIC (*fC*max/MIC) and the area under the free concentration-time curve over 24 h divided by the MIC (*f*AUC/MIC) (Nakamura et al., 2019). The dosing regimen is essential for the reaching of required exposure value for the bacteriostatic and bactericidal effect. In clinical trials, dosing regimen of cefidercol that implies 2 g in every 8 h through 3-h infusion) achieves *f*T > MIC of 100%, for MIC ≤4 μg/ml (Katsube et al., 2014).

Utilized humanized regimen of cefidercol in rat lung infection model with 3 –h infusion reached higher %fT _MIC_ (100%) and better efficiency against carbapenem-resistant *P. aeruginosa*, *A. baumannii*, and *K. pneumoniae* strains than 1 -h infusion (70% fT _MIC_) suggesting that larger exposure time leads to better effectiveness of cefidercol (Matsumoto et al., 2017). All isolates with MIC ≤4 μg/ml showed similar susceptibility to cefidercol in comparison with isolates with MIC>4 suggesting MIC of 4 μg/ml as a breakpoint for cefidercol after using the humanized dosing regimen of 24 h in a neutropenic murine thigh model.

#### Preclinical Studies: *in vitro*, *in vivo*

##### *In vitro* Studies

Antibacterial activity of cefiderocol determined *in vitro* and expressed as MIC values largely depend on the concentration of ferric iron in the medium on which the activity is tested due to the unique mechanism of antibiotic penetration by chelating ferric ions. Therefore, iron-depleted cation-adjusted Mueller-Hinton broth (ID-CAMHB) prepared with Chelex^®^ 100 resin that mimics a physiological state of reduced iron concentration in acute infections has been approved for determination of cefiderocol antibacterial potency in broth microdilution or disk diffusion methods (Huband et al., 2017). The MICs breakpoints for cefiderocol published by CLSI against Enterobacteriaceae, *P. aeruginosa*, *Acinetobacter* species, and *S. maltophilia* are ≤4 μg/ml (susceptible), 8 μg/ml (intermediate), and ≥16 μg/ml (resistant) (Wayne, 2011). Recently, FDA has published lower MICs break points than CLSI for *E. coli*, *K. pneumoniae*, *Proteus mirabilis* and *E. cloacae* (2 μg/ml, 4 μg/ml, 8 μg/ml) and *P. aeruginosa* (≤1 μg/ml, 2 μg/ml, ≥4 μg/ml) (Food and Drug Administration, 2019).

The first report of cefiderocol antibacterial activity against 617 clinical Enterobacteriaceae isolates demonstrated its high antibacterial potential against *E. coli*, *K. pneumoniae*, *Serratia marcescens*, *Citrobacter freundii*, *Enterobacter aerogenes*, and *E. cloacae* isolates with MIC_90_ values of ≤1 μg/ml regardless of species and only 8 isolates (1.3%) had MICs ≥8 μg/ml. Additionally, cefiderocol had excellent antibacterial activity against 226 characterized β-lactamase producing strains and only seven isolates producing NDM-1 (5), VIM (1), and IMP (1) carbapenemases had MICs ≥16 μg/ml (Kohira et al., 2016). Cefiderocol was also active against 316 non-fermenting Gram-negative bacilli belonging to *A. baumannii*, *P. aeruginosa* and *S. maltophilia* showing MICs_90_ of 2, 1 and 0.5 μg/ml, respectively. MDR strains of *A. baumannii* and *P. aeruginosa* including strains with identified β-lactamases were also inhibited by cefiderocol. CR strains of *A. baumannii* possessing β-lactamases (IMP-1, OXA-23, OXA-24, OXA-51/ISAba1 or OXA-58) had cefiderocol MIC_90_ of 8 mg/ml. *P. aeruginosa* strains with characterized βlactamases (GIM-1, IMP, SPM-1 and VIM) were also susceptible to cefiderocol with MIC_90_ of 4 μg/ml (Ito et al., 2016a).

The significant antibacterial potential of cefiderocol was further confirmed through the global surveillance studies of cefiderocol activity conducted on a collection of clinical Gram-negative bacilli in North America and Europe collected in the 2014–2015 (SIDERO-WT-2014) and 2015–2016 (SIDERO-WT-2015) period.

Comparative agents (cefepime, ceftazidime-avibactam, ceftolozane-tazobactam, ciprofloxacin, colistin and meropenem) were also used for testing bacterial susceptibility. Results from SIDERO-WT programs revealed that cefiderocol MICs were very low for the most of tested isolates, and >99% of the isolates were susceptible to cefiderocol in each testing period (Hackel et al., 2017; Karlowsky et al., 2019). Among Enterobacteriaceae, only 17 of 12 100 isolates had cefiderocol MICs >4 μg/ml, and 14 of them were inhibited at a concentration of 8 μg/ml. Cefiderocol was active against 99, 9% of *P. aeruginosa*, 97,6% of *A. baumannii* in the first study and 96,4% *Acinetobacter* spp. isolates in the second testing period at a concentration of ≤4 μg/ml. The most resistant isolates were found in *Acinetobacter* spp group. cefiderocol also demonstrated superior antibacterial activity against *S. maltophilia* and Burkholderia spp. isolates with MIC_90_ values 4- to 64- fold lower than those of comparativeagents. Compared with the other tested agents, cefiderocol showed better antibacterial activity, except for meropenem against Enterobacteriaceae, while ceftazidime-avibactam against Enterobacteriaceae had the same or less MIC_90_ compared to cefiderocol in both studies. Colistin was the second active agent against *P. aeruginosa* and *Acinetobacter* spp. isolates. In the SIDERO-WT-2016 program, 10 470 isolates were tested in 2016–2017 period. In comparison with results from the previous testing period, cefiderocol MIC_90_ values were higher for *Citrobacter koseri* and *A. baumannii* isolates, and the isolates from tree new tested species (*Morganella morganii*, *Proteus vulgaris*, *Proteus mirabilis*) had low values of cefiderocol MIC_90_ (Yamano, 2019).

The global surveillance studies further showed that cefiderocol was superior to six comparative agents in its antibacterial activity against meropenem-non-susceptible clinical isolates of Gram-negative bacilli.

Only colistin had similar MIC_90_ as cefiderocol against *P. aeruginosa* isolates, while the MICs for other agents were 4, 8, and 64-fold higher compared to cefiderocol MIC_90_ for all tested groups. Cefiderocol MIC_90_ ranges for Enterobacteriaceae, *P. aeruginosa* and *Acinetobacter* spp. meropenem-non-susceptible isolates were 1–4, 0.5–1, 1–2 μg/ml, respectively.

Excellent antibacterial activity of cefiderocol against carbapenem-non-susceptible and MDR Gram-negative bacilli were demonstrated in the SIDERO-CR-2014/2016 program, where 96.2% of all tested isolates were susceptible to cefiderocol (Hackel et al., 2018).

Cefiderocol demonstrated the best *in vitro* antibacterial activity against MDR *P. aeruginosa*, and *S. maltophilia* isolates with MIC_90_ values of 1 and 0.25 μg/ml, respectively, while MIC_90_ for carbapenem-non-susceptible Enterobacteriaceae was 4 μg/ml. The highest cefiderocol MIC_90_ was obtained for MDR *A. baumannii* isolates (8 μg/ml) among which 89.7% (330/368) were nonsusceptible to cefiderocol and with the strains having MICs of 256 μg/ml. Slight differences in susceptibilities of isolates to cefiderocol were noticed concerning the geographical distribution of the isolates in North America and Europe. (Supplementary Table 1).

Antibacterial *in vitro* activity of cefiderocol also was evaluated in small-scale studies in different countries. All CR *A. baumannii* and Enterobacteriaceae isolates collected from Greek hospitals were susceptible to cefiderocol (MICs ≤1 μg/ml), while only 13% imipenem-resistant *P. aeruginosa* and *A. baumannii* strains isolated from patients with bacteremia in Taiwan had cefiderocol MICs ≥4 μg/ml (Falagas et al., 2017; Hsueh et al., 2019). CR strains of KPC-possessing *K. pneumoniae*, *P. aeruginosa* and *A. baumannii* showed cefiderocol MIC_90_ values 2-to 8- fold higher than the surveillance isolates from the same species in research conducted in New York City (Iregui et al., 2020). High-risk clones of MDR Enterobacteriaceae, *A. baumannii*, *P. aeruginosa* and *S. maltophilia* collected in Spain were susceptible to cefiderocol in a high percentage (98%) (Delgado-Valverde et al., 2020). Among 478 Gram-negative bacilli isolated from cancer patients, belonging to Enterobacteriaceae (including CR and ESLB producing strains), *P. aeruginosa* (including MDR strains), *Acinetobacter* spp., *S. maltophilia*, *Burkholderia cepacian*, and some uncommon bacteria such as Pantoea spp., *Sphingomonas paucimobilis*, *Elizabethkingia meningoseptica* and *Rhizobium radiobacter*, 97,5% were susceptible to cefiderocol. Results obtained from Whole-genome sequencing of 12 non-susceptible isolates revealed that *Klebsiella* spp. isolates had disruption of outer membrane porins OmpK36, OmpK37, and OmpK35 and the various β-lactamases, *Enterobacter* spp. isolates demonstrated alterations in OmpC and OmpF and had AmpC and ESBLs, while carbapenemases and various β-lactamases were present among *Acinetobacter* spp. isolates (Rolston et al., 2020).

Cefiderocol antibacterial activity against carbapenem-non-susceptible strains with defined resistance mechanisms was examined to find a correlation between cefiderocol MICs and the production of β-lactamases. Molecular identification of β-lactamase genes in the strains collected in SIDERO-WT-2014 project revealed that 67% of tested isolates were β-lactamases producers. KPC, VIM, NDM, OXA-48 and GES types of enzymes were found in Enterobacteriaceae and cefiderocol showed the highest MIC_90_ (8 μg/ml) against NDM producers. *P. aeruginosa* isolates harboring VIM, IMP or GES type β-lactamases had cefiderocol MICs ≤2 μg/ml, while cefiderocol exhibited MICs >8 μg/ml against 21 of 667 *A. baumannii* isolates carrying OXA-23 and OXA-24 genes (Kazmierczak et al., 2019).

Jacobs et al. reported that cefiderocol MIC_90s_ in Enterobacteriaceae depended on β lactamases types. The groups harboring ESLBs (TEM and SHV), NDM, and KPC-2 β-lactamases had the highest MIC_90_ of 8 μg/ml, followed by KPC-3 producers with MIC_90_ of 2 μg/ml and strains with OXA-48-like lactamase with MIC_90_ of 1 μg/ml.

On the other hand, the activity of cefiderocol against non-fermenting bacilli wasn’t affected by the presence of β lactamases including carbapenemases. Cefiderocol had excellent activity against *P. aeruginosa* strains carrying bla_VIM_, bla_PDC_ and porin OprD genes and L1 producers of S*. maltophilia* strains with MIC_90_ being 0.5 and 0.25 μg/ml, respectively. The cefiderocol MIC_90_ against *A. baumannii* complex, producing OXA types βlactamases was 1 μg/ml (Jacobs et al., 2019). Analysis of cefiderocol MICs by regression analysis showed that cefiderocol antibacterial activity was not significantly affected by expression of genes encoding porins or efflux systems in *K. pneumoniae*, *P. aeruginosa* and *A. baumannii*. There was no correlation between cefiderocol MIC values and increased expression of ampC in *P. aeruginosa* and *A. baumannii*. *A. baumannii* isolates possessing ESBLs had significantly higher cefiderocol MICs than isolates without ESBLs but the presence of OXA23-did not affect the antibacterial activity of cefiderocol (Iregui et al., 2020).

Cefiderocol demonstrated potent activity against carbapenem-non-susceptible Enterobacteriaceae including ESBLs and AmpC producers of *E. coli* and *K. pneumonia*, as well as carbapenem-non-susceptible and MDR *P. aeruginosa*, *S. maltophilia* and *A. baumannii* strains isolated from patient in Canadian intensive care units. Production of ESBLs (CTX- M-type, TEM-15, SHV) was confirmed by molecular methods for most of isolates. All isolates (800) were susceptible to cefiderocol and the highest cefiderocol MICs (4 μg/ml) were detected in the *K. pneumoniae* SHV-producer isolates and an ESBL phenotype which wasn’t confirmed by molecular characterization. (Supplementary Table 2).

##### *In vivo* Studies

After estimated *in vitro* efficiency against clinically important Gram-negative bacteria, cefiderocol profile was examined in thigh and lung animal infection models with Gram-negative bacteria, including CR strains of Enterobacteriaceae, *P. aeruginosa*, *A. baumannii* and *S. maltophilia*. Review of preclinical data showed that the efficacy of cefiderocol in different types of infection, as well as against MDR and CR strains, was similar (Supplementary Table 3).

Cefiderocol was efficient against Gram-negative bacilli (19 strains of Enterobacteriaceae*, P. aeruginosa, A. baumannii* and *S. maltophilia*) including CR strains in the thigh and lung neutropenic murine infection models (Nakamura et al., 2019). In the thigh infection model, mean %fT > MIC required for bactericidal and bacteriostatic effect was similar for different species, but there was significant difference between CR strains and susceptible strains (*p 0.045) indicated that CR strains required higher %fT > MIC of cefiderocol for a 1-log_10_ reduction than carbapenem-susceptible strains.

In lung infection model, cefiderocol demonstrated similar efficacy as in thigh infection model, 64.4 ± 22.5% for reduction of 9 strains of Enterobacteriaceae and 70.3 ± 9.0% for reduction of 3 strains of *P. aeruginosa.* Mean values %fT > MIC required for 1-log_10_ reduction of 3 *A. baumannii* (88.1 ± 3.4%) and 4 *S. maltophilia* strains (53.9 ± 18.1%) were slightly different and there was the need for larger sample to find the exact cause. *Monogue et al.* used a larger sample of Gram-negative isolates (95) including MDR strains in neutropenic murine thigh infection model (Monogue et al., 2017). Results of this study predict that for the Enterobacteriaceae, *A. baumannii*, and *P. aeruginosa* isolates with cefidercol MIC ≤4 μg/ml, %fTMIC of 96.2% is required for bacterial reduction after humanized 24 h-exposition to cefiderocol. In this group of isolates, the results of bacterial density study showed similar number of isolates that showed bacterial reduction or stasis. Isolates with MICs ≥8 μg/ml did not have consistent results, showing reduction or stasis in 2 of 28 isolates (7%) and growth reduction in 13 of 22 isolates (59%). It was noticed that 2 *A. baumannii* strains and 1 strain of *K. pneumoniae* showed changes in pre and postexposure MICs indicated the need to examine changes in MICs after the period of 72 h because of the previously observed resistance in siderophore antibiotics (Kim et al., 2015).

Cefiderocol had similar efficacy as meropenem and cefepime against 15 selected Enterobacteriaceae*, A. baumannii, and P. aeruginosa* isolates but exerted mean bacterial reduction of 1.5 ± 0.4 log_10_ CFU at 24 h for meropenem and cefepime resistant strains. 12 MDR isolates (2 *P. aeruginosa*, 4 *A. baumannii*, 6 Enterobacteriaceae) were chosen for prolonged 72 h study in neutropenic murine thigh infection model (Stainton et al., 2019). Humanized cefiderocol exposures over 72 h revealed that cefiderocol had sustained antimicrobial activity against most MDR isolates including cefepime-resistant strains. No adaptive response was determined, except for one *E. coli* 462 strain that showed 4-fold postexposure MIC increase.

Cefiderocol demonstrated better efficacy compared to cefepime against *P. aeruginosa* SR27016 in the thigh murine infection model. It was shown that %fT > MIC was a parameter best correlated with *in vivo* cefiderocol activity with values—%fT > MIC 61.7% for bacteriostatic effect and %fT > MIC 87 for 1-log10 reduction (Nakamura et al., 2019) Higher mean fT > MIC values were reported in testing cefiderocol activity against 8 *P. aeruginosa* isolates, that have previously showed different response to the other siderophore β-lactam antibiotics (Ghazi et al., 2018b). Mean %fT > MIC required for stasis was 76.3 ± 18.4, 81.9 ± 18.3 for 1 log_10_ reduction and 88.2 ± 15.9 for 2 log_10_ CFU reduction and cefiderocol had better *in vivo* activity compared to two siderophore β-lactams, MB-1 and SMC-3176. as well as cefepime and levofloxacin (Ghazi et al., 2018a).

In immunocompetent-rat respiratory tract infection model, *Matsumoto et al.* confirmed that the %fT > MIC is the most important PD parameter for indicating *in vivo* cefiderocol effectiveness (Matsumoto et al., 2017). Activity of cefiderocol under humanized dosing regimen was determined against 6 CR strains of *P. aeruginosa, A. baumannii*, and *K. pneumoniae*, with different infusion period from1 to 3 h. Higher reduction in bacterial number was observed after 3 h infusion ranging from 3.04 log_10_ CFU to 4.41 log_10_ than in 3h infusion with a range of 0.7 log_10_ CFU to 3.7 log_10_ CFU. Prolonged infusion time led to increasing %fTMIC, as shown with other lactam antibiotics. For the isolates with MICs of 4 μg/ml, %fT MIC was 70% after 1 h infusion and 100% after prolongated infusion. The isolates with lower MICs achieved the same %fT MIC of 100% in both regimens. (Supplementary Table 3).

### Therapeutic Efficacy—Clinical Studies, Phase II and Phase III Clinical Trials

Therapeutic efficacy and safety of cefiderocol have been evaluated through three completed clinical trials. The results obtained in phase II clinical trial APEKS-cUTI (NCT02321800) have been published in a peer-review journal, while the results from phase III trials CREDIBLE-CR (NCT02714595) and APEKS-NP (NCT03032380) are only available through Shionogi reports and scientific conference announcements.

A phase II trial GAMECHANGER (NCT03869437) has recently started to examine the efficacy of cefiderocol compared to the best available therapy (BAT) for the treatment of bloodstream infections caused by Gram-negative pathogens (Health, 2018). Summary of the results from clinical studies is presented in Table 1.

**TABLE 1:** Clinical studies.

ClinicalTrials.gov ID, status, locations	Study design	No of patients in mITT	Dosing regiment	Primary outcome		Secondary outcome		Interpretation	Ref
Phase II trials									
APEKS-cUTI NCT02321800, Completed, United States, Belgium, Canada, Czechia, Estonia, France, Georgia, Germany, Hungary, Japan, Russian Federation, Serbia, Spain, Taiwan, Ukraine	Treatment of cUTI with or without pyelonephritis or AUP	FDC: 252	2 g (t′ 1 h) q8 h 7–14 days	Composite of clinical response and microbiological response at TOC in the mITT population [% (n/N)]		Microbiological response per patient at EA/EOT/TOC/FUP (%)		FDC was superior to high dose IPM/CIS. Cefiderocol is safe and effective for the treatment of cUTI.	ClinicalTrials.gov (2014); Portsmouth et al. (2018)
						FDC: IPM/CIS:	92.1/96.8/73.0/57.1 90.8/95.8/56.3/43.7		
		IPM/CIS: 119	1 g/1 g (t′ 1 h) q8 7–14 days	FDC: 72.6 (183/252)		Microbiological response per pathogen at EA/EOT/TOC/FUP (%)			
				IPM/CIS: 54.6 (65/119)					
	Multicenter, double-blind, parallel-group non-inferiority trial								
						*E. coli* (152, 79)			
		Total: 371				FDC:	92.8/98.7/75.0/59.9		
						IPM/CIS:	94.9/97.5/58.2/41.8		
						*K. pneumoniae* (48, 25)			
						FDC:	89.6/97.9/75.0/58.3		
						IPM/CIS:	88.0/92.0/52.0/52.0		
						*P. eruginosa* (18, 5)			
						FDC:	94.4/88.9/44.4/27.8		
						IPM/CIS:	80.0/100/60.0/20.0		
						*P. mirabilis* (17, 2)			
						FDC:	88.2/94.1/76.5/64.7		
						IPM/CIS:	100/100/50.0/0		
						Clinical response per patient at EA/EOT/TOC/FUP (%)			
						FDC:	90.5/98.0/89.7/81.3		
						IPM/CIS:	90.8/99.2/87.4/72.3		
						Clinical response per pathogen (%)			
						*E. coli* (146, 77)			
						FDC:	91.8/97.9/89.7/82.9		
						IPM/CIS:	96.1/98.7/88.3/72.7		
						*K. pneumoniae* (46, 25)			
						FDC:	82.6/100/89.1/82.6		
						IPM/CIS:	88.0/100/84.0/68.0		
						*P. eruginosa* (15, 4)			
						FDC:	93.3/93.3/73.3/53.3		
						IPM/CIS:	75.0/100/75.0/75.0		
						*P. mirabilis* (13, 1)			
						FDC:	84.6/100/100/84.6		
						IPM/CIS:	100/100/100/100		
GAMECHANGERNCT03869437, In progress, Australia, Greece, Italy, Singapore, Thailand, Turkey	Treatment of BSI	FDC	2 g (t′ 3 h) q8 h 14 days	All-cause mortality at day 14				Health (2018); ClinicalTrials.gov (2019)	
	Multicenter, randomized, open-label trial	BAT Total 284	Chosen by the investigator 14 days						
Phase III trials									
CREDIBLE-CR NCT02714595, Completed, United States, Brazil, Croatia, France, Germany, Greece, Guatemala, Israel, Italy, Japan, Korea, Thailand, Turkey, United Kingdom	Treatment of HAP, VAP, HCAP, cUTI or BSI/sepsis	FDC: 80	2 g (t′ 3 h) q8 h 7–14 days	Clinical outcome per patient at TOC for HAP/VAP/HCAP, BSI/sepsis, cUTI		All-cause mortality at day 14, day 28 and day 49 % Day 14, Day 28/Day 49		Microbiological eradiation in cUTI subgroup was higher in FDC group than BAT group Higher mortality rates were detected in the FDC group than in the BAT group for HAP/VAP/HCAP and BSI/sepsis subgroups	FDA (2019a)
		Clinical cure (% (n/N))							
	Multicenter randomized, open-label	BAT: 38 Total 118	Chosen by the investigator 7–14 days	Overall		Overall			
				FDC:	52.5 (42/80)	FDC:	18.8/24.8/33.7		
				BAT:	50.0 (19/38)	BAT:	12.2/18.4/20.4		
				HAP/VAP/HCAP		HAP/VAP/HCAP			
				FDC:	50.0 (20/40)	FDC:	24.4/31.1/42.2		
				BAT:	52.6 (10/19)	BAT:	13.6/18.2/18.2		
				BSI/sepsis		BSI/sepsis			
				FDC:	43.5 (10/23)	FDC:	16.7/23.3/36.7		
				BAT:	2.9 (46/14)	BAT:	5.9/17.6/23.5		
				cUTI		cUTI			
				FDC:	70.6 (12/17)	FDC:	11.5/15.4/15.4		
				BAT:	60.0 (3/5)	BAT:	20/20/20:		
				Microbiological outcome per patient at TOC for cUTI, HAP/VAP/HCAP, BSI/sepsis Eradication (% (n/N))		All-cause mortality at day 49 by baseline pathogen [% (n/N)]			
				cUTI		*A. baumannii*			
				FDC:	52.9 (9/17)	FDC:	48.7 (19/39)		
				BAT:	20.0 (1/5)	BAT:	23.5 (4/17))		
				Overall		*K. pneumoniae*			
				FDC:	31.3 (25/80)	FDC:	23.5 (8/34)		
				BAT:	23.7 (9/38)	BAT:	25.0 (4/16)		
				HAP/VAP/HCAP		*P. eruginosa*			
				FDC:	22.5 (9/40)	FDC:	35.3 (6/17)		
				BAT:	21.1 (4/19	BAT:	16.7 (2/12)		
				BSI/sepsis		*S. maltophilia*			
				FDC:	30.4 (7/23)	FDC:	80.0 (4/5)		
				BAT:	28.6 (4/14)	BAT:	0.0 (0/0)		
APEKS-NPNCT03032380, Completed, United States, Belgium, Canada, Czechia, Estonia, France, Georgia, Germany, Israel, Japan, Latvia, Philippines, Puerto Rico, Russian Federation, Serbia, Spain, Taiwan, Ukraine	Treatment of nosocomial pneumonia, including HAP, VAP and HCAP Multicenter, randomized, double-blind parallel-group	FDC 148	2 g (t′ 3 h) q8 h 7–14 days	All-cause mortality at day 14% (nN)		Clinical outcome at TOC Clinical cure %		FDC was non-inferior to high dose MEM.	Health (2018); Wunderink et al. (2019)
		MEM 150	2 g (t′ 3 h) q8 h 7–14 days	Day 14 FDC: MEM: Day 28 FDC: MEM:	Day 28 12.4 (18/145)11.6 (17/146)21.020.5	FDC: MEM: Microbiological eradication at TOC % FDC: MEM:	64.8 66.7 7.6 48		
			LZD in each group 600 mg (t′ 30 min–2 h) q12 h ≥5 days	Mcrobiologically evaluable per protocol population FDC: MEM:	13.0 (13/100)	Clinical cure rates per pathogens at TOC *K. pneumoniae* FDC: MEM: *E. coli*	64.6 (31/48) 65.9 (29/44)		
		Total: 298				FDC:	63.2 (12/19)		
						MEM:	59.1 (13/22)		
						*P. eruginosa*			
						FDC:	66.7 (16/24)		
						MEM:	70.8 (17/24)		
						*A. baumannii* FDC: MEM:	52.2 (12/23) 58.3 (14/24)		

UTI, complicated urinary tract infection; AUP, uncomplicated pyelonephritis; HAP, hospital acquired pneumonia; VAP, ventilator associated pneumonia; HCAP, healthcare-associated pneumonia; BSI, bloodstream infections; FDC, cefiderocol; IPM/CIS, imipenem/cilastatin; MEM, meropenem; LZD, linezolid; BAT, best available therapy; EA, early assessment; EOT, end of treatment; TOC, time of cure; FUP, follow-up; mITT, modified intention-to-treat.

In APEKS-cUTI study, cefiderocol was compared to imipenem-cilastatin for the treatment of the patients with complicated urinary tract infection (cUTI), with or without pyelonephritis or acute uncomplicated pyelonephritis (AUP) (Portsmouth et al., 2018). The patients who met the study criteria (448) were randomized 2:1 to receive cefiderocol or imipenem-cilastatin. After excluding patients who had Gram-negative uropathogenic counts ≤1 × 10⁵ CFU/ml from initial groups, 252 patients in the cefiderocol group and 119 in the imipenem-cilastatin group were further enrolled in the modified intention-to-treat (mITT) population. In the imipenem-cilastatin group, the percentage of patients with acute uncomplicated pyelonephritis was higher than in the cefiderocol group for 3%. The most isolated Gram-negative pathogens from the baseline urine cultures were *E. coli* and *K pneumoniae* which were equally presented in both groups, while *P aeruginosa* was more isolated from the patients in the cefiderocol group (7%) than in the imipenem-cilastatin group (3%).

The primary outcome of the trial was the composite of clinical and microbiological response at the test of cure (TOC) assessment, 5–9 days after antibiotic treatment was finished. The primary endpoint efficiency was 72.6% (183/252) in the cefiderocol group and 54.6% (65/119) in the imipenem/cilastatin group with a difference of 18.58% (95% CI 8.·23 to 28.·92; *p* = 0·0004) implying non-inferiority of cefiderocol compared to imipenem/cilastatin at the specified 20 and 15% margins. Subsequent post-hoc analyses revealed that cefiderocol was superior to high dose imipenem/cilastatin.

Secondary outcomes were clinical and microbiological responses per-pathogen and per-patient at different time points: early assessment (EA)−4 ± 1 days from beginning, end of treatment (EOT)−7 to 14 days from the start, TOC−7 ± 2 days after the end of treatment and follow-up (FUP)-approximately 14 days after end of treatment (follow-up), as well as safety testing.

Similar microbiological responses in both groups were determined at EA and EOT. At TOC and FUP, cefiderocol had better efficiency compared to imipenem/cilastatin in microbiological eradication, with differences between groups of 17·25% (95% CI 6.·92–27.·58) and 13·92% (95% CI 3.·21–24.·63), respectively. On the other hand, clinical responses were similar between the two groups at EA, EOT and TOC, while the sustained clinical response in cefiderocol group at FUP was better for 9.·02% (95% CI −0.·37–18.·41) in comparison with imipenem/cilastatin group. At TOC per-pathogen, the composite outcome for *P aeruginosa* was similar between groups, 47% in the cefiderocol group and 50% in the imipenem-cilastatin groups but lower than the overall response rate.

The better composite response in the cefiderocol group was achieved for *E coli* and *K pneumoniae* than in the imipenem-cilastatin group, with differences between groups of 16 and 26%, respectively. The composite response for ESBLs producing pathogens at TOC was 63% for the cefiderocol group and 47% for the imipenem-cilastatin group with the difference of 16.66%.

Mild and moderate AEs including diarrhoea, hypertension, constipation, infusion site pain, headache, nausea, hypokalaemia, renal cyst, insomnia, infusion site erythema, abdominal pain upper, cardiac failure, *Clostridium difficile* colitis, and vaginal infection occurred more frequently in the imipenem-cilastatin group (76 of 148 patients-51%) than in the cefiderocol group (122 of 300 patients-41%).

Among severe AEs reported in 5% patients in the cefiderocol group and 8% patients in the imipenem-cilastatin group, *C difficile* colitis was the most common.

A phase III clinical trial (CREDIBLE-CR) was conducted to compared cefiderocol efficiency for the treatment of severe infections caused by CR Gram-negative pathogens to other antibiotics representing BAT (FDA, 2019a).

It was a multicenter, randomized, open-label clinical study enrolling the patients with clinically documented hospital-acquired pneumonia (HAP), ventilator-associated pneumonia (VAP), healthcare-associated pneumonia (HCAP), bloodstream infections (BSI), sepsis, and cUTI. BAT was consisted of up to three antibiotics, with colistin included in 66% regimens and was administrated intravenously by country-specific guidelines. Of 150 randomly selected patients with diagnosed diseases, 118 with confirmed CR pathogens were chosen for the mITT population, 80 in cefiderocol group and 38 in BAT group. A. baumannii, *K. pneumoniae* and *P. aeruginosa* were the most often isolated baseline pathogens.

The primary endpoint was clinical outcome per patient at TOC, 7 days after the end of the treatment, as well as microbiological outcome per patient at TOC for the patients with cUTI. At TOC, overall clinical cure rates were similar between groups, 52.5% in the cefiderocol group and 50% in the BAT group, as well as for the subgroups defined by clinical diagnosis (Table 1). Microbiological eradiation in cUTI subgroup was higher for 32.9% (95% CI −9.4–75.3) in the cefiderocol group than in the BAT group.

One of the secondary outcomes was the determination of all-cause mortality (ACM) at Day 14, Day 28 and Day 49. Higher mortality rates were detected in the cefiderocol group than in the BAT group for HAP/VAP/HCAP and BSI/sepsis subgroups at all timepoints, the highest difference was in HAP/VAP/HCAP subgroup at Day 49, 24.0% (95% CI − 2.4–45.7). ACM was lower in cUTI subgroup at all time points but these results are difficult to interpret due to the insufficient number of patients in the group and wide confidence interval. Cefiderocol groups also had higher mortality rates at Day 49 in the patients infected with *A. baumannii* (cefiderocol group 49% (19/39) vs. BAT group 24% (4/17)) and *P. aeruginosa* (cefiderocol group 35% (6/17) vs BAT group 17% (2/12). All patients with *S. maltophilia* infections were in the cefiderocol group, with 80% (4/5) mortality at day 49. Analysis of PK in patients who survived (57) and died (22), revealed that deaths were not associated with cefiderocol exposure. MICs of baseline pathogens were increased a 4-fold in 15 patients treated with cefiderocol and in 5 patients in the BAT group, fatal infection mortality was 60% for cefiderocol group and 20% in the BAT group. Increased mortality in cefiderocol group could be also appeared because of adjunctive therapy. Infection-related death with treatment failure was 7.7% more common in cefiderocol group than in the BAT group. Septic shock, pneumonia, sepsis, and bacteremia were the most common treatment-emergent adverse events (TEAEs) connected to mortality.

Therapeutic efficacy of cefiderocol was compared to meropenem for the treatment of nosocomial pneumonia caused by Gram-negative pathogens in phase III APEKS-NP clinical study (Job, 2019; Wunderink et al., 2019).

The patients (298) with clinically diagnosed HAP, VAP and HCAP were randomized (1:1) and received combined therapy of cefiderocol or meropenem with linezolid included for the treatment of Gram-positive bacteria. The primary outcome was ACM at Day 14 in the mITT population. The difference between groups at Day 14 was 0.8% (95% CI-6.6–8.2) in favor of cefiderocol confirming non-inferiority of cefiderocol to high dose meropenem at the specified 12.5% margin. At TOC, clinical outcome and microbiological eradication were similar between groups, and clinical cure rate among the most frequently isolated pathogens was higher in the cefiderocol group only for *E. coli*, difference 4.1% (95% CI-25.8–33.9). TEAEs frequency was 87.8% (130/148) in the cefiderocol group and 86.0% (129/150) in the meropenem group.

### Dosage and Administration, Tolerability

Cefiderocol requires intravenous administration as its sulfate tosylate salt due to its high molecular weight and poor oral bioavailability. It is approved and marketed under the brand name Fetroja^®^ in the US, Japan, and the European Union.

The therapeutic dose to maintain antibiotic effectiveness for a period of 24 h above the minimum inhibitory concentration in patients with normal renal function is 2 g infused over 1 h administered every 8 h (Katsube et al., 2017b). The half-life of cefiderocol in healthy adults is 2.74 h with a c_max_ of 156 μg/mL reached within 1 h (t_max_) following administration (Saisho et al., 2018). The antibiotic is cleared at a rate of 5.13 L/h and the fraction cleared unchanged is 61.5%. Within the tested dose range (100–2,000 mg) cefiderocol exhibited linear first-order pharmacokinetics. Another study traced radioactively labelled cefiderocol following intravenous administration and recovered the total dose after 120 h, primarily in urine (90.6% was unchanged cefiderocol out of 98.2% total radioactivity) (Miyazaki et al., 2019). Cefiderocol also accounted for 92.3% of the radioactivity in plasma, indicating minimal metabolism.

Dose adjustment is necessary for cefiderocol in patients with renal impairment (Katsube et al., 2017b). All patients receive the drug as an infusion over at least a one-hour time period. While patients with normal (CL_Cr_ 90 < 120 mL/min) or mildly impaired (CL_Cr_ 60 < 90 mL/min) kidney function receive 2 g every 8 h, lower doses of 1.5 g are required for patients with moderate (CL_Cr_ 30 < 60 mL/min) and further reduction to 1 g for severe (CL_Cr_ 15 < 30 mL/min) kidney impairment. Both patients with end-stage renal disease or on intermittent hemodialysis should only receive 0.75 g cefiderocol every 12 h to avoid adverse effects. In addition to adjusting the dosing interval, the infusion duration can be changed from 1 h to 3 h to further reduce the risk of adverse outcomes or potential toxic blood concentrations. In cases of pyelonephritis with a CL_Cr_ > 120 mL/min, the dosing interval can be adjusted to 2 g every 6 h to compensate for increased glomerular filtration rate because of acute kidney inflammation (Kawaguchi et al., 2018).

Drug-drug interactions of cefiderocol have not been reported to date.

The antibiotic presents with a moderate protein binding of 58% thus not interfering with highly protein-bound drugs (Matsumoto et al., 2017). Human drug transporter interactions were evaluated in healthy volunteers following co-administration with respective substrates for the organic anion transporter (OAT1/3), the organic cation transporter and multidrug and toxin extrusion (OCT1/2, MATE-2K), and organic anion transporting polypeptide (OATP1B3) (Katsube et al., 2018b). The plasma concentrations of the substrates were not significantly affected by intravenous co-administration with cefiderocol although the substrate for the OATP1B3 showed elevated plasma levels that were not regarded as clinically significant.

Safety and tolerability data from clinical trials indicate that cefiderocol has a similar profile to other cephalosporin antibiotics (Saisho et al., 2018).

In both single- and multiple-dose administration studies, major reported adverse effects were diarrhea, rash, local injection site reactions, pyrexia, and elevated liver enzyme levels (alanine and aspartate aminotransferases, respectively). Cefiderocol does not affect the QT interval in therapeutic and supratherapeutic (up to 4 g) doses in healthy adult subjects (Sanabria et al., 2019). Although mild to moderate antibiotic-associated diarrhea can be a common adverse effect, there is also a risk of *Clostridium difficile* infection and related severe diarrhea linked to the antibiotic cefiderocol treatment course (Portsmouth et al., 2018).

Cefiderocol could be associated with a higher risk of death in patients hospitalized with other severe bacterial infections (such as pneumonia or sepsis) aside from the cefiderocol indicated complicated urinary tract infection. The cause of death could therefore not be causatively linked to cefiderocol use or other bacterial infections (FDA, 2019b).

### The Place of Cefiderocol in the Treatment of Serious Gram-Negative Bacterial Infections

The clinical efficacy of cefiderocol has been proven in several cases of severe infections that have been successfully treated with cefiderocol.

Compassionate use of cefiderocol was in the instances where other conventional therapeutic options were inefficacious or if serious AEs connected with applied antibiotic therapy occurred.

Cefiderocol was used as adjunct therapy with colistin for treatment of healthcare-associated native aortic valve endocarditis caused by extremely drug-resistant (XDR) *P. eruginosa* carrying bla (Vietnam ESBL) gene and lacking OprD porin (Edgeworth et al., 2019).

Recurrent bacteremia due to MDR *P. aeruginosa* in the patient with left ventricular assist devices was cured after inclusion of cefiderocol in current therapy with meropenem and tobramycin (Sigmon et al., 2020).

As antipseudomonal monotherapy, cefiderocol was used in the treatment of an intraabdominal infection caused by MDR *P. aeruginosa*. The patient, in life-threatening condition after receiving aminoglycosides and polymyxin antibiotics, was discharged from hospital after 28 days of cefiderocol administration (Stevens and Clancy, 2019).

Cefiderocol monotherapy also led to a rapid improvement in the condition of the critically ill patient with VAP and BSI caused by XDR *A. baumannii* and KPC-producing *K. pneumoniae* (Trecarichi et al., 2019). Prolongated use of cefiderocol for 95 days was documented in the treatment of implant-associated chronic osteomyelitis in a 15-year old adolescent with XDR *P. aeruginosa* carrying blaNDM-1 and *K. pneumoniae* producing ESBL β-lactamase (Alamarat et al., 2019). Initial therapy consisted of cefiderocol and aztreonam led to an inCRase in liver function, so aztreonam was discontinued and the patient was treated only with cefiderocol to the end of the therapy.

Osteomyelitis caused by XDR *A. baumannii* with 12 molecular characterized resistance genes was successfully treated for 109 days with cefiderocol in combination with daptomycin for treatment of *E. faecalis* and *Corynebacterium striatum* which also were isolated from the patient specimen (Dagher et al., 2020). Acute osteomyelitis was successfully treated with cefiderocol in combination with ceftazidim/avibactam and colistin in the patient with carbapenemases producing strains of *A. baumannii* (OXA-23), *E. cloacae* (KPC) and *P. aeruginosa* (VIM) (Zingg et al., 2020). The same research team reported two more cases. The patient with postoperative implant-associated infection of the spine caused by *A. baumannii* harboring OXA-40 and NDM carbapenemases was recovered under the treatment with cefiderocol, ceftazidim/avibactam and colistin. Pleural empyema due to *A. baumannii* strain producing OXA-23 and OXA-58 carbapenemases was suppressed with cefiderocol and colistin.

The episodes of acute neutropenia in the last days of the therapy which was resolved after the discontinuation of the treatment and decrease in white cell count resolved spontaneously were the only AEs reported (Alamarat et al., 2019; Edgeworth et al., 2019). One fatal outcome was presented for a patient who developed multiple infections after kidney transplantation. Cefiderocol in combination with polymixin B and ceftazidim/avibactam was used against two strains of *K. pneumonia* carrying bla_NDM-1_, bla_OXA-232_, bla_CTX-M-15_, armA, and tet(D) genes isolated from blood and the abdominal cavity. However, it was not possible to determine precisely how cefiderocol contributed to this negative outcome due to polymicrobial infections with vancomycin-resistant enterococci, *Candida glabrata* and *Clostridoides difficile* (Contreras et al., 2020).

## Conclusion

Therapeutic superiority of cefiderocol to high dose imipenem/cilastatin for the treatment of cUTI and AUP was demonstrated in phase II clinical trial. Based on these results, the FDA approved cefiderocol for the treatment of adults with cUTI, including kidney infections, when other therapeutic options are limited.

Therapeutic efficacy of cefiderocol was evaluated for the treatment of HAP, VAP and HCAP in two studies within the phase III clinical trials. In APEKS-NP clinical study, cefiderocol was non-inferior to a high dose of meropenem for the treatment of nosocomial pneumonia, but further investigations are needed for proving the possible role of cefiderocol in the treatment of these serious infections. In an ongoing study, the efficacy of cefiderocol compared to BAT for the treatment of BSI caused by Gram-negative pathogens is examined. A few clinical case reports have provided additional insight into possible clinical uses of cefiderocol, such as osteomyelitis that were successfully treated in three patients by prolongated cefiderocol administration.

Cefiderocol already has an important place in the treatment of severe urinary tract infections caused by Gram-negative bacilli. Data on the possible use of cefiderocol in the treatment of nosocomial pneumonia and BSI are still limited, but future studies through clinical trials and case reports will provide an answer.
