# Supplementary material for: The Burden of the Serious and Difficult-to-Treat Infections and a New Antibiotic Available: Cefiderocol
Source: Front Pharmacol. 2021 Jan 14;11:578823. doi: 10.3389/fphar.2020.578823 (PMC7898678; doi:10.3389/fphar.2020.578823)
Supplement: Supplementary file 1 [file DataSheet1_v1.PDF]

Table 1. In vitro antibacterial activity of Cefiderocol against Gram-negative bacilli in global surveillance studies

	SIDERO-WT-2014 Study (Hackel et al., 2017)			SIDERO-WT-2015(Karlowsky et al., 2019)		
**Family/genus/species**	**No. of isolates**	**MIC range (μg/ml)**	**Susceptibility (%)[a]**	**No. of isolates**	**MIC range (μg/ml)**	**Susceptibility (%)[a]**
Enterobacteriaceae	6087	≤0.002–8	99.9%	6013	≤0.002–128	99.9%
MEM-non-susceptible	169	0.008–8	97.0%	246	0.008-8	99.6%
Klebsiella spp.	2031	≤0.002–8		1981	≤0.002–4	
K. pneumoniae	1526	≤0.002–8	99. 6 %	1528	≤0.002–4	
K. oxytoca	505	≤0.002–2		389	≤0.002–2	
K. variicola				35	≤0.002–2	
E. coli	1529	≤0.002–4		1830	≤0.002–8	99.9 %
Serratia spp.	996	≤0.002–8		794		
Se. marcescens	927	≤0.002–8	99.8%	776	0.015-32	99.7 %
Se. liquefaciens	33	0.015–0.25				
Enterobacter spp.	1024	0.004-8		933		
E. aerogenes	442	0.004–8	99.8%	244	≤0.002-4	
E. cloacae	514	0.008–4		594	≤0.002-128	99.7 %
E. asburiae	30	≤0.06–0.5		40	≤0.002–4	
Citrobacter spp.	507	≤0.002–4		475		
C. freundii	303	≤0.002–2		252	≤0.002–8	99.6 %
C. koseri	172	0.008–4		169	0.015-8	99.4 %
P. aeruginosa	1530	≤0.002–8	99.9%	1540	≤0.002–8	99.9%
MEM-non-susceptible	353	≤0.002–4		395	≤0.002–8	99.7%
Acinetobacter spp.				972	≤0.002–>256	96.4%
MEM-non-susceptible				562	≤0.002–>256	96.1%
A. baumannii	1148	≤0.002–64	97.6%	837	≤0.002–>256	
MEM-non-susceptible	768	≤0.002–64	96.9%)			
A. pitii				111	≤0.002–1	
S. maltophilia	428	≤0.002–4		340	≤0.002-64	99.4%
B. cepacia	12	0.015-16	93.8%			
B. cepacia complex				89	≤0.002–32	94.4%

MEM-non-susceptible	31	≤0.002–32	87.1%

SIDERO-CR-2014/2016 (Hackel et al., 2018)

**Family/genus/species**	**No. of isolates**	**MIC range (µg/ml)**	**MIC_{50} (µg/ml)**	**MIC_{90} (µg/ml)**	**Susceptibility (%)[a]**
Enterobacteriaceae	1022	0.004-32	1	4	97
E. coli CNS	73	0.015-4	1	2	
K. pneumoniae CNS	689	0.004-32	1	4	98,3[b]
Enterobacter spp. CNS	158	0.06-32	2	8	88.6[b]
Se. marcescens CNS	39	0.015-4	0.5	2	
Citrobacter spp. CNS	32	0.015-8	0.5	2	96.9[b]
K. oxytoca CNS	31	0.03-4	0.25	1	
A. baumannii MDR	368	0.015->256	0.25	8	89.7
P. aeruginosa MDR	262	≤0.002-32	0.25	1	99.2
S. maltophilia	217	0.004-2	0.06	0.25	
B. cepacia	4	0.004-8			

[a] Susceptible isolate with MICs ≤4□g/ml, [b] non-susceptible isolates with MIC ≥8□g/ml, CNS-carbapenem non-susceptible, MDR-multidrug resistant isolates

Table 2. In vitro antibacterial activity of cefiderocol against Gram-negative bacilli in different studies

Family/genus/species	No. of isolates	MIC range (µg/ml)	MIC_{50} (µg/ml)	MIC_{90} (µg/ml)	Susceptibility (%)[a]	Comparative agents	Ref
E. coli	106	≤0.063-4	0.125	1		CAZ, FEP,	(Kohira et
β-lactamase-positive	78	≤0.125->16			94[b]	MEM, LVX	al., 2016)
K. pneumoniae	105	≤0.063-2	0.5	0.125			
β-lactamase-positive	81	≤0.125-2					
Se. marcescens	103	≤0.063->64	≤0.063	≤0.063	95[b]		
C. freundii	100	≤0.063->64	≤0.063	0.125	99[b]		
E. aerogenes	100	≤0.063->8	0.063	0.5	99[b]		
E. cloacae	103	≤0.063->16	0.125	1	99[b]		
A. baumannii	104	≤0.063-4	0.125	2		CAZ, FEP, LVX,	(Ito et al.,
β-lactamase-positive	29	0.03-32	0.5	8	90[b]	MEM, PIP/TAZ	2016a)
P. aeruginosa	104	≤0.063-4	≤0.063	1			
β-lactamase-positive	33	0.03–8	0.5	4	97[b]		
S. maltophilia	108	≤0.063–4	0.125	0.5			
E. coli	164	0.03-64	0.5	4		AMK, ATM,	(Dobias et
K. pneumoniae	298	0.03-64	1	2		CAZ, CIP, CST,	al., 2017)
Enterobacter spp.	159	0.03-64	0.5	4		CT CZA, MEM,	
P. aeruginosa	45	0.03-64	0.5	2		FEP, TGC	
carbapenemases-positive	30		0.5	2			
A. baumannii	87	0.03-64	0.12	4			
OXA- positive	85		0.12	4			
Enterobacteriaceae							
KPC- positive	127	0.03-64	1	2			
OXA-48- positive	154	0.03-64	0.25	2			
NDM, VIM, IMP-positive	134		1	4			
K. pneumoniae	244	≤0.03-4	0.5	1		CAZ, CZA, FEP,	(Falagas et
E. cloacae	14	0.06-4	0.5	1		MEM	al., 2017)

Pr. stuartii	11	0.06-0.5	0.25	0.5			
A. baumannii	107	≤0.03-2	0.06	0.5			
P. aeruginosa	82	≤0.03-2	0.12	0.5			
Enterobacteriaceae	834	≤0.03->64	0.5	4	91.5	AMK, ATM, CAZ, CIP, CST, C/T, CZA, FEP, MEM, TGC	(Jacobs et al., 2019)
None	18	≤0.03-4	≤0.03	≤0.03			
KPC-2-positive	355	≤0.03-32	1	8			
KPC-3-positive	380	≤0.03-64	0.25	2			
KPC-4, KPC-4-like-positive	2	0.15-16	0.5	16			
NDM-positive	28	0.25->64	2	8			
OXA-48-like-positive	7	≤0.03-1	0.25	1			
NDM, OXA-48-like-positive	1	1					
Other (TEM ESBL, SHV ESBL, CTX-M, PER, and/or AmpC)-positive	43	≤0.03->64	2	8			
A. baumannii	200	≤0.03->64	0.12	1	97		
CNS	99	≤0.03->64	0.12	0.5	97.9		
CR	101	≤0.03->64	0.25	1	96		
P. aeruginosa							
VIM and PDC-positive	27	≤0.0-1	0.25	0.5			
S. maltophilia							
L1 and L2-positive	25	≤0.03-0.25	0.06	0.25			
A. baumannii						AMK, ATM, CAZ, CIP, CST, C/T, CZA, FEP, MEM, TGC	(Hsueh et al., 2019)
IPM-resistant	100	0.06-> 64	0.5	8	88[b]		
P. aeruginosa	100	≤ 0.03-8	0.12	1	99[b]		
IPM- resistant	25	≤ 0.03-2	0.06	0.5			
MEM- resistant	75	≤ 0.03-8	0.12	2	98.7[b]		
S. maltophia	100	≤ 0.03-1	0.06	0.25			
E. coli	1869	≤ 0.03-2	0.12	0.5			(Iregui et al., 2020)
Enterobacter spp.	172	≤ 0.03-1	0.12	0.5			

K. pneumoniae	517	≤ 0.03-2	0.12	0.5		CAZ, CIP, GEN,	
CR	111	≤ 0.03-4	1	2		MEM, PIP/TAZ	
A. baumannii	46	0.06-4	0.25	1		TMP/SMX,	
CR	78	0.12->32	0.5	8	88		
P. aeruginosa	269	≤ 0.03-8	0.25	0.5	99.6		
CR	130	≤ 0.03-4	0.5	1			
E. coli	177	≤0.03-2	≤0.03	0.5		CST, C/T, CZA, MEM, PIP/TAZ	(Golden et al. 2020)
ESBL- positive	29	≤0.03-2	0.25				
AmpC- positive	6	≤0.03-2	≤0.03				
K. pneumoniae	121	≤0.03-4	≤0.03	0.25			
ESBL-positive	11	≤0.03-4	1				
K. oxytoca	46	≤0.03-0.5	≤0.03	0.12			
K. aerogenes	24	≤0.03-0.5	0.06				
E. cloacae	93	≤0.03-2	0.12	1			
Se. marcescens	47	≤0.03-1	≤0.03	0.25			
Pt. mirabilis	14	≤0.03-0.25	≤0.03				
Enterobacterales, ETP non-susceptible	21	≤0.03-4	0.25				
A. baumannii	11	≤0.03-0.25	0.06				
P. aeruginosa	201	≤0.03-2	0.06	0.5			
MEM- non-susceptible	54	≤0.03-1	0.12	1			
MDR	29	≤0.03-1	0.12				
S. maltophilia	66	≤0.03-4	0.12	0.5			
K. pneumoniae	121	≤0.03-4	0.5	2		AMK, ATM, CAZ, CIP, CST, C/T, CZA, FEP, MEM, TGC	(Delgado-Valverde et al., 2020)
ST11/OXA-48+CTX-M-15	25	≤0.03-4	0.25	2			
ST15/OXA-48+CTX-M-15	25	≤0.03-4	0.25	4			
ST512/KPC-3	25	0.25-4	2	4			
ST258/KPC-3	25	0.06-4	2	2			
ST147/OXA-48	3	0.06-0.5	0.25	0.5			

ST392/OXA-48+CTX-M-15	4	0.06-1	0.25	0.1			
E. cloacae	4	0.5-8			50		
A. baumannii	80	0.06-16	0.25	4	95		
P. aeruginosa	6	0.125-0.5					
S. maltophilia	20	≤0.03-2	0.25	0.5			
E. coli ESBL-positive	52	<0.03-4	ND	2		AMK, ATM, CAZ, CIP, CST, C/T, CZA, FEP, MEM, TGC	(Rolston et al., 2020)
K. pneumoniae-positive	37	0.125-64	ND	2	97		
CR Enterobacteriaceae	20	0.06->64	ND	4	95		
Citrobacter spp.	20	<0.03-8	ND	1	95		
E. cloacae	38	<0.03->64	ND	4	90		
Serratia spp.	20	<0.03-0.5	ND	0.5			
Acinetobacter spp.	20	<0.03->64	ND	4	90		
P. aeruginosa MDR	32	<0.03->64	ND	1	97		
S. maltophilia	50	<0.03-4	ND	0.5			

[a] Susceptible isolate with MICs ≤4□g/ml, [b] non-susceptible isolates with MIC ≥8□g/ml, CR-carbapenem resistant, CNS-carbapenem non-susceptible, IPM resistant-imipenem resistant, MEM resistant-meropenem resistant, ETP non-susceptible- ertapenem non-susceptible amikacin-AMK, amoxicillin-clavulanic acid-AMZ, aztreonam-ATM, cefepime-FEP, ceftazidime-CAZ, ceftazidime–avibactam-CZA, ceftolozane/tazobactam-C/T, ceftriaxone-CRO, ciprofloxacin-CIP, colistin-CST, gentamicin-GEN, levofloxacin-LVX, meropenem-MEM, piperacillin/tazobactam-PIP/TAZ, tazobactam-CT, tigecycline-TGC, trimethoprim/sulfamethoxazole-TMP/SMX

Table 3. Antibacterial activity of cefiderocol in animal models.

Family/species/ strains	Dosing regimen	n(R)[a]	MIC (μg/ml)	%fTMIC Bactericidal effect	%fTMIC Bacteriostatic effect	Isolates with bacterial stasis (%)	Ref
**Neutropenic murine thigh infection model**							
Enterobacteriaceae	0.2-ml s.c injections, simulated to have %fTMICs as in human exposure	31				77	(Monogue et al., 2017)
A. baumannii		16	≤4	96.20		88	
P. aeruginosa		20				85	
Enterobacteriaceae		8					
A. baumannii		19	≥8			7.14	
P. aeruginosa		1					
P. aeruginosa	humanized exposures 15, 20, 25, 10, and 5 mg/kg administered at 0, 1, 2, 4, and 6 h, q8h	8	0.063-0.5			100[1]<br>87.5[2]	(Ghazi et al., 2018a)
P. aeruginosa	subcutaneously at doses of 4.2, 8.3, 16.7, 33.3, 66.7, 100.0, 133.3 and 166.7 mg/kg, q8h	8					(Ghazi et al., 2018b)
PSA 1403			0.063	97[3]<br>99.5[4]	93.6		
PSA 1401			0.25	57.6[3]<br>65[4]	53.3		
PSA JJ8-16			0.125	92.7[3]<br>98.2[4]	86.7		
PSA JJ5-35			0.25	87.9[3]<br>94.1[4]	82.5		
PSA AZ8-18			0.5	50.2[3]<br>62.1[4]	44.4		
PSA AZ32-13			0.25	79.9[3]<br>86.9[4]	74.4		

PSA JJ4-36			0.125	97.5[3]<br>99.9[4]	94.7	
PSA JJ11-54			0.25	92.5[3]<br>100[4]	81.1	
Enterobacteriaceae	to 600 mg/kg q3h, 2 h postinfection, 24-h period			73.3 ± 23.3	62.5±27.4	(Nakamura et al., 2019)
E. coli			0.125			
K. pneumonia			0.125-16			
P. aeruginosa		13(7)	0.25-2	72.2± 21.4	63.0± 15.5	
P. aeruginosa SR27016	24, 80, 240, 800 mg/kg, sc- administered, q24h, q12h, q6h, q3h, 24h period		0.25	57.60	47.50	
**Murine lung infection model**						
Enterobacteriaceae	0.1 to 600 mg/kg q3h, 2 h postinfection, 24-h period	19(7)		64.4±22.5		(Nakamura et al., 2019)
E. coli			4			
K. pneumoniae			2-16	70.3±9.0		
P. aeruginosa			0.5-2	88.1±3.4		
A. baumannii			0.25-2	53.9±18.1		
S. maltophilia			0.125-0.25			
**Immunocompetent-Rat Respiratory Tract Infection Model**						
P. aeruginosa<br>*A. baumannii*<br>*K. pneumoniae*	2 g q8h, as a 1-h infusion and 3 h infusion, 96 h	6(5)	0.125-8	75 [5]<br>100[6]		(Matsumoto et al., 2017b)

Abbreviations and symbols: [a]n(R)- Total isolate number (number of carbapenem or MDR resistant), [1]≥1 log_{10} reduction; [2]≥2 log_{10} reduction, [3] - 1-log10 reduction; [4]- 2-log_{10} reduction; after 1-h infusion; [6] after 3-h infusion
